# Substance use disorders among Aboriginal and Torres Strait Islander people in custody: a public health opportunity

**DOI:** 10.1186/s40352-016-0044-8

**Published:** 2016-12-05

**Authors:** Ed Heffernan, Fiona Davidson, Kimina Andersen, Stuart Kinner

**Affiliations:** 1 School of Medicine, Brisbane, Queensland Australia; 2 Metro North Health and Hospital Services, Brisbane, Queensland Australia; 3 Griffith Criminology Institue and Menzies Health Institue, Brisbane, Queensland Australia

**Keywords:** Indigenous population, Prisons, Alcohol, Drugs, Mental disorders

## Abstract

**Background:**

To describe the prevalence, type, and mental health correlates of substance use disorders in a large sample of incarcerated Indigenous Australians.

**Methods:**

An epidemiological survey of the mental health of Indigenous people in custody in the state of Queensland, Australia was conducted using culturally informed methods. The prevalence, type and mental health correlates of substance use disorders were determined using a diagnostic interview and questionnaire.

**Results:**

In a sample of 396 Indigenous people (331 males, 65 females) the prevalence of any substance use disorder was 66%. Alcohol dependence (males 47%, females 55%) was the most common type of substance use disorder, followed by cannabis dependence (males 20%, females 26%). Mental illness (anxiety, depression and psychotic disorder), and lifetime suicide thoughts and attempts, were significantly more likely among those with a substance use disorder. The majority of the sample reported intoxication with alcohol (70%) and/or other drugs (51%) at the time of arrest. Most individuals (87%) had not accessed alcohol and other drug services in the 12 months prior to custody.

**Conclusions:**

Substance dependence was common in this sample and was associated with other forms of mental health adversity, yet most individuals reported no access to health services prior to incarceration. Effectively responding to substance dependence for Indigenous Australians is a public health and criminal justice priority. Culturally capable alcohol and other drug treatment services in custody and in the community are critical, and should be co-located and coordinated with mental health services.

## Background

The 2015 Australian Medical Association report card on the health Aboriginal and Torres Strait Islander people (Indigenous Australians) described significant concern over the escalation in incarceration rates (Australian Medical Association 2015). The report interpreted this problem as symptomatic of the health disparity between Indigenous and non-Indigenous Australians. The age standardised rate of incarceration for Indigenous Australians is 13 times that of non-Indigenous Australians (Australian Bureau of Statistics 2014) and the majority of Indigenous people in custody suffer from mental disorders (Heffernan et al. 2012; Australian Institute of Health and Welfare 2015). Substance use disorders in particular are key drivers of this high incarceration rate (Weatherburn 2008; NIDAC 2009; Krieg 2006) and a significant contributor to the health burden and health inequality of Indigenous Australians (Vos et al. 2009; Holland 2014). Substance use in Indigenous Australians has negative impacts on physical (Australian Institute of Health and Welfare 2011), social and community well-being (National Indigenous Drug and Alcohol Council 2014). The Australian National Mental Health Commission described the high rates of incarceration of Indigenous people as “shocking” (National Mental Health Commission 2013) and noted the relationship with the high prevalence of mental disorder in this group. These concerns were also highlighted in a recent annual report that monitors Australia’s progress in addressing the health gap between Indigenous and non-Indigenous Australians (Holland 2015).

While the custodial setting offers an opportunity (albeit a regrettable one) to access treatment for health-related problems, poor health outcomes post-release including relapse to risky substance use and the elevated risk of preventable morbidity and mortality (Kinner et al. 2011; Alan et al. 2011; Kariminia et al. 2006; Borschmann et al. in press) suggest that this rarely translates into sustained health improvements. A key challenge in reversing these poor public health and criminal justice outcomes is ensuring adequate access to culturally appropriate alcohol and other drug (AOD) treatment services for prisoners (National Indigenous Drug and Alcohol Council 2014) and for the same individuals after they return to the community.

A recent study of prison entrants in Australia identified just how prevalent problematic substance use is for both Indigenous and non-Indigenous prisoners. However, it also highlighted the need for different service responses to address these challenges for Indigenous people (Doyle et al. 2015). One critical first step in developing such services is understanding patterns of AOD service utilisation before incarceration and the extent and types of substance use diagnosis in this population. This understanding could help inform service design to better meet the needs of Indigenous Australians. We undertook further analysis of data from Australia’s largest systematic study of mental disorders amongst Indigenous people in custody (Heffernan et al. 2012) to describe the prevalence and type of substance use disorder amongst a sample of Indigenous men and women in Queensland custody, and to explore the associations between substance use disorder and other mental disorders, arrest and community health service utilisation in this group prior to incarceration.

## Methods

The methodology for this study was a cross sectional survey using a standardised diagnostic instrument and a questionnaire (Heffernan et al. 2012).

### Participants

Surveys were conducted in six of the nine correctional centres in the state of Queensland, these were selected as they housed the highest proportion of indigenous prisoners and included remanded and sentenced prisoners and all levels of security classification. The six targeted centres held approximately 75% of Indigenous males and 90% of Indigenous females incarcerated (either remanded in custody (pre-trial) or sentenced) in Queensland at the time. Potential participants were identified from the nominal role on the first day that the researchers visited that centre. From this role every female (100%) and every third male (33%) who self-identified as Indigenous (Aboriginal, Torres Strait Islander or both) were approached to participate in the study. Excluded from the sample were those judged unable to provide informed consent and those considered too physically or mentally unwell to participate.

### Measures

Data were collected via face-to-face interviews in confidential settings within the custodial centres. Prisoners were provided with information about the survey in verbal and written form to ensure that they understood the purpose and voluntary nature of participation. Interviews were conducted by Indigenous researchers with mental health experience, who were trained in the use of the research tools, ethical and emergency care procedures. The research assistants were supported by an Indigenous manager and clinician, and a psychiatrist was available if required. The study relied on the involvement of Indigenous people in the design, implementation, data collection and interpretation of results, and was informed by a comprehensive community consultation process (Queensland Government 2009).

Assessments were made via a questionnaire, a diagnostic instrument and, where indicated, clinical interviews for diagnosing psychotic disorders. The questionnaire covered demographic, social, custodial, mental health, health care and cultural characteristics. Participants were asked about their history of suicide thoughts and acts (current, past 12 months, lifetime), and about utilisation of AOD services and services for mental health care (psychiatrist, psychologists, general practitioner, inpatient or community mental health services or counsellor) in the 12 months prior to custody.

The Composite International Diagnostic Instrument (CIDI) version 2.1 (World Health Organization 1997) was administered to assess participants for depression and anxiety during the previous 12 months, and substance use disorders during the 12 months prior to custody. Although the CIDI has not been validated for an Australian Indigenous population, it is a comprehensive and fully standardised interview (World Health Organization 1997) that has been validated internationally in numerous cultures and languages. In addition, it was chosen because Indigenous mental health experts and Indigenous mental health workers who examined and trialled the tool considered the depression, anxiety, and substance use disorder modules to be culturally appropriate for this population, and the CIDI has been used widely with Indigenous populations in other large prisoner studies (Butler et al. 2005) and in major mental health surveys in Australia (Slade et al. 2009). In addition to cater for an incarcerated population we modified the standard CIDI questions for substance use disorders from the past 12 months to the 12 months before incarceration, and used ICD-10 criteria for harmful use and dependence (World Health Organization 1993).

### Data analysis

Data were analysed using Stata v13.0 (StataCorp 2013); descriptive statistics are reported, and Odds Ratios (ORs) with 95% confidence intervals (95%CI) are reported for comparisons between those with and without a substance use disorder (Table 1). Further analysis for associations between substance use disorders and variables in Table 1 was done using a backward stepwise multivariate logistic regression analysis. Statistically significant associations are reported in the Results section.

**Table 1:** Demographic characteristics and odds of mental illness according to substance use disorder

	No SUD (*n* = 134)		SUD (*n* = 262)		OR (95% CI)
	*n*	%	*n*	%	
Age in years					
< 30	59	44	147	56	
30+	75	56	115	44	1.63 (1.05–2.53) *p* = 0.023
Gender					
Female	20	15	45	17	
Male	114	85	217	83	1.18 (0.67–2.10) *p* = 0.568
Current Relationship	60	45	126	48	1.14 (0.75–1.74) *p* = 0.532
Education <year 10	70	52	162	62	1.48 (0.97–2.26) *p* = 0.067
Custody Status^a^					
Remanded	36	27	98	40	
Sentenced	94	70	158	60	1.61 (1.02–2.56) *p* = 0.037
Adult Incarceration^a^					
1–3	75	57	129	51	
4 or more	56	43	126	49	1.31 (0.86–2.00) *p* = 0.215
Mental illness	35	26	100	38	1.75 (1.10–2.76) *p* = 0.017

^a^missing data *n* = 10

## Results

### Sample

During the survey period there were 1381 Indigenous males (mean age 28.8 years) and 116 Indigenous females (mean age 30.5 years) in custody in Queensland. Of 487 males approached to participate in the study, 347 (71%) were interviewed, 92 declined to participate, 45 were released, transferred or not available and 3 were judged too unwell to be seen due to mental illness. Of the 88 females approached to participate in the study 72 (82%) were interviewed; 10 declined to participate, 5 were released and 1 was judged too physically unwell to be seen. Among the final sample of 419 individuals, 396 (95%) completed the diagnostic interview and were included in this study. Of the 396 participants, 81% identified as Aboriginal, 8% as Torres Strait Islander and 12% as both. The male participants (mean age 31.5 years) were on average 2.7 years older than male non-participants (mean age = 28.8 years) (*p* = 0.03). There was no statistically significant difference in the age of female participants (mean age = 29.2 years) and non-participants (mean age = 30.5 years) (*p* > 0.05).

### Demographic characteristics

The majority of participants were male (84%), not in a relationship (53%), had less than 10 years of education (59%) and had been in custody on more than 1 occasion (81%). One third (34%) had at least one mental illness (anxiety, depressive or psychotic disorder). Those with a substance use disorder were on average significantly younger, more likely to be on remand, and more likely to have a mental illness than those without a substance use disorder (Table 1).

### Substance use disorders

**Fig. 1:**
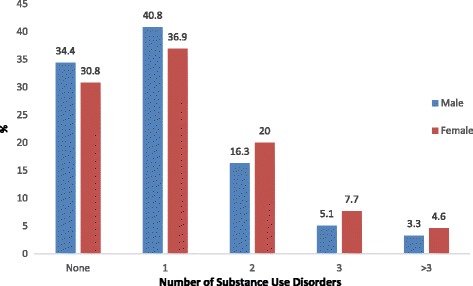
Co-occurring substance use diagnoses by gender

Two thirds of participants (66%) had at least one substance use disorder, almost always including substance *dependence* (63%). The most prevalent substance use disorder for both men and women was alcohol dependence (males 47%, females 55%), followed by cannabis dependence (males 20%, females 26%). There were no significant differences in the prevalence of substance use disorders between males and females (all *p* > 0.05) (Table 2). A high proportion of both males (25%) and females (32%) had multiple, co-occurring substance use disorders (Fig. 1). Those with a substance use disorder were significantly more likely to be less than 30 years of age (37 vs. 29%, *p* = 0.02), to be on remand (pre-trial detention) (40 vs. 27%, *p* = 0.04) and to have a mental disorder (38 vs. 26%, *p* = 0.02). Associations for potential explanatory variables (Table 1) with substance use disorders were examined using multivariate logistic regression analysis. The associations between substance use disorder and age less than 30 (AOR = 1.60, 95% CI 1.05–2.44, *p* = 0.03) and mental illness (AOR = 1.72, 95% CI 1.09–2.73, *p* = 0.02) remained statistically significant (LR chi2(2) = 10.7).

**Table 2:** Prevalence of substance use disorder according to sex

ICD 10 Diagnosis	Male (*n* = 331)	Female (*n* = 65)	Total (*N* = 396)
	*n* (%)	*n* (%)	*n* (%)
Alcohol	170 (51.3)	39 (60.0)	209 (52.7)
Dependence	155 (46.8)	36 (55.3)	191 (48.2)
Harmful Use	15 (4.5)	3 (4.6)	18 (4.5)
Amphetamine	36 (10.8)	4 (6.1)	40 (10.1)
Dependence	34 (10.2)	4 (6.1)	38 (9.6)
Harmful Use	2 (0.6)	0 (0.0)	2 (0.5)
Cannabis	70 (21.1)	17 (26.1)	87 (21.9)
Dependence	66 (19.9)	17 (26.1)	83 (20.9)
Harmful Use	4 (1.2)	0 (0.0)	4 (1.0)
Opioids	32 (9.6)	7 (10.7)	39 (9.8)
Dependence	32 (9.6)	7 (10.7)	39 (9.8)
Harmful Use	0 (0.0)	0 (0.0)	0 (0.0)
Sedatives	6 (1.8)	4 (6.1)	10 (2.5)
Dependence	0 (0)	1 (1.5)	1 (0.2)
Harmful Use	6 (1.8)	3 (4.6)	9 (2.2)
Others^a^	28 (8.4)	11 (16.9)	39 (9.9)
Dependence	24 (7.2)	11 (16.9)	35 (8.8)
Harmful Use	4 (1.2)	0 (0.0)	4 (1.0)
Any Substance	217 (65.5)	45 (69.2)	262 (66.1)
Dependence	208 (62.8)	43 (66.1)	251 (63.3)
Harmful Use	23 (6.9)	3 (4.6)	26 (6.5)

^a^Others = hallucinogens, volatile substances, stimulants other than amphetamines

### Behavioural and health service correlates of substance use disorder

Selected behavioural and health service correlates of substance use disorder are shown in Table 3. At the time of the incident that led to their arrest the majority of participants (males 70%, females 66%) reported being under the influence of alcohol and just over half (males 51%, females 51%) reported being under the influence of illicit drugs, including cannabis (70%), amphetamines (38%) and opioids (20%). Those with a substance use disorder were significantly more likely than those without a SUD to be under the influence of alcohol (21 vs. 49% *p* = 0.03) or other drugs (13 vs. 38% *p* < 0.001) at the time of their arrest. Only a minority of participants reported accessing AOD treatment services (13%) or mental health care (23%) in the 12 months prior to custody. Those with a substance use disorder were significantly more likely than those without a substance use disorder to have accessed AOD services (15 vs. 8% *p* = 0.03). Finally, those with a substance use disorder were significantly more likely than those who did not have a substance use disorder to have had lifetime suicide thoughts (OR = 1.68, 95%CI 1.03–2.78, *p* = 0.03) and to have attempted suicide at some point in their life (OR = 1.91, 95%CI 1.05–3.61, *p* = 0.03) .

**Table 3:** Associations between suicidality, intoxication at the time of arrest, service access, and substance use disorder

	No SUD (*n* = 134)		SUD (*n* = 262)		OR (95% CI)
	*n*	%	*n*	%	
Suicidal Thoughts and Attempts					
Thoughts (Lifetime)	33	25	93	36	1.68 (1.03–2.78) *p* = 0.028
Thoughts (12mths)	12	9	31	12	1.36 (0.65–3.02) *p* = 0.383
Thoughts (Current)	2	2	6	2	1.55 (0.27–15.86) *p* = 0.592
Attempt (Lifetime)	18	13	60	23	1.91 (1.05–3.61) *p* = 0.025
Under Influence of substance at the time of arrest					
Alcohol	84	63	192	73	1.63 (1.02–2.60) *p* = 0.029
Drugs^a^	50	37	150	57	2.25 (1.44–3.53) *p* < s 0.001
Service access in the 12 months pre custody					
Mental Health Care^b^	29	22	58	22	1.03 (0.61–1.78) *p* = 0.906
AOD Service	10	8	40	15	2.23 (1.05–5.18) *p* = 0.027

^a^missing data *n* = 3 (counted as not intoxicated)

^b^missing data *n* = 15 (10 with SUD)

## Discussion

### Key findings

The challenges of substance misuse among Aboriginal and Torres Strait Islander people in Australian custody have been highlighted previously. However, this study has, for the first time, articulated the prevalence, type, co-occurrence and correlates of clinical substance use disorder diagnosis in a systematically surveyed cohort. Substance dependence is highly prevalent (males 63%, females 66%) and substantially more prevalent than hazardous use in this cohort; this is the reverse of what is found in the general Australian community (Australian Institute of Health and Welfare 2014a). This finding is consistent with suggestions of a causal link between substance dependence and incarceration for Indigenous Australians (National Indigenous Drug and Alcohol Council 2014). Alcohol dependence was the most prevalent substance disorder (males, 47% and females, 55%). This has significant clinical implications for health services in custody, which are responsible for both the acute management of substance withdrawal and the longer-term management of recovery and rehabilitation.

Another key finding from this study was the high prevalence of cannabis dependence, with one in five males and over a quarter of females diagnosed as dependent. Co-occurring substance use disorders were also common, with more than a quarter of the sample having two or more substance use disorders. In addition, a substantial proportion had a mental illness, either a psychotic, mood or anxiety disorder (males 29%, females 60%) and, consistent with findings from community studies (Slade et al. 2009), mental illness was significantly more common amongst those with a substance use disorder. Similarly, while the prevalence of suicide ideation and attempts were high in this group, lifetime suicide thoughts and attempts were significantly more common in those with a substance use disorder. This highlights the complex mental health needs of this group and illustrates the critical importance of providing integrated, culturally informed mental health and substance use care, both in custody and after return to the community.

Our findings also support the role of substance misuse as a significant contributor to the disproportionate incarceration rate for Indigenous people. The majority of individuals in our study reported being under the influence of alcohol and approximately half reported being under the influence of illicit drugs, most often cannabis, at the time of offending. Importantly, those with a substance use disorder were significantly more likely to report intoxication at the time of their arrest. Coupled with well-established evidence of substance-related morbidity and mortality after release from custody (Kinner et al. 2011; Forsyth et al. 2014; Winter et al. 2015) it seems logical that a focus on the provision of adequate and culturally appropriate drug and alcohol interventions would not only serve as an important public health intervention, but assist in reducing the incarceration (and re-incarceration) rate of Indigenous people (National Indigenous Drug and Alcohol Council 2014; Kinner and Wang 2014).

This study highlights the critical need to ensure adequate drug and alcohol and mental health treatment for Indigenous Australians in custody. The return to risky substance use after release from custody is common and predictable (Kinner 2006; Thomas et al. 2013), as is the high rate of mortality and morbidly of Indigenous Australians following release from custody (Kinner et al. 2011; Alan et al. 2011; Forsyth et al. 2014). A recent national review of supply, demand and harm reduction strategies in Australian prisons identified limitations in culturally appropriate approaches to drug and alcohol services for Indigenous Australians and no substantial focus on continuity of care into the community (Rodas et al. 2011). The latest annual snapshot of publicly funded alcohol and drug treatment services in Australia provide no information on what is provided for Indigenous Australians in custody (Australian Institute of Health and Welfare 2014b). The striking lack of information on culturally competent AOD services for this population stands in stark contrast to the recognition of this as a priority health area for the national campaign to close the health gap between Indigenous and non-Indigenous Australians (Holland 2016). There is knowledge, evidence and proposals about how to achieve success in this area (National Indigenous Drug and Alcohol Council 2014; Gray et al. 2014) and it is timely that this becomes a public health priority in Australia.

### Strengths and limitations

Key strengths of this study include the efforts undertaken to ensure that the methodology was culturally appropriate, the relatively large and representative sample, and the use of well-validated assessment tools. One challenge of research in custodial settings is achieving systematic sampling, given the rapid flow of people in and out of custody on a daily basis. We attempted to address this by surveying centres that contained the majority of Indigenous prisoners and we estimated that we interviewed 25% of all males and 62% of all females. Nevertheless, we cannot guarantee the generalisability of this sample to Indigenous prisoners across Australia. Although this is the largest systematic study of an incarcerated Australian Indigenous population, we only sampled participants from the state of Queensland, which accounted for 28% of Australia’s Indigenous prisoner population at the time of the study (Australian Bureau of Statistics 2009). It would be prudent to replicate our research in other Australian jurisdictions, however the health profile of Indigenous prisoners is strikingly similar across the country (Australian Institute of Health and Welfare 2013), suggesting that our findings are likely to have national and potentially international relevance.

Another potential limitation of our study relates to use of the CIDI. Our rationale for selecting this tool is articulated in the methods section, and we consider it unlikely that the CIDI would pose a significant risk of culturally-based measurement bias. Although there is a risk of recall bias associated with applying the CIDI to the 12 months before prison, rather than the past 12 months, we consider it unlikely to have substantially affected the findings given that the majority of participants had been in custody for less than a year (males 59.2%, females 75.1%).

## Conclusions

This study has described important, additional clinical information, enhancing the understanding of the treatment needs for substance use problems amongst Indigenous Australians in custody. This information has included; the prevalence of dependence, the limited access to appropriate health services prior to custody, and the significant associations between substance use disorders, mental disorder, suicidality and offending in this cohort. These problems are costly, not only to individuals, but also to communities, public health and criminal justice systems (National Indigenous Drug and Alcohol Council 2014; Alan et al. 2011; Thomas et al. 2013; Australian Institute of Health and Welfare 2013; Heffernan et al. 2009). Despite advances in knowledge around treatment approaches (National Health and Medical Research Council 2009) and the fact that uptake of health care in prisons by Indigenous Australians is usually better than in the community (Australian Institute of Health and Welfare 2013) there has been limited progress in reducing post-release substance-related mortality, morbidity and re-incarceration. However, there have been significant advances in the understanding of what is needed in the delivery of alcohol and other drug services to Aboriginal and Torres Strait Islander peoples (Gray et al. 2014). This includes cultural capability, continuity of care, the need for Indigenous leadership and holistic and integrated services that are strengths based. The critical next step in this challenge is not further evidence about the size of the problem, but rather delivery of culturally secure services that are resourced appropriately, at a scale commensurate with need and rigorously evaluated using culturally informed methodologies.
